# The Royal Hospital of the Innocents

**Published:** 1902-08-16

**Authors:** 


### THE ROYAL HOSPITAL OF THE INNOCENTS.

On entering the Piazza of the most Holy Annunciation from the Via Ricasoli, the graceful arcades forming the loggia of the Ricasoli Foundling Hospital proclaim the design of a master mind. The silk mercers of Florence in the year 1419 determined to make further provision than already existed for the reception and charge of foundlings, and entrusted the design to Filippo di Ser Brunellesco. The building gradually grew up and was completed in 1444, its architectural grace being further adorned by some of the finest specimens of Lucca della Robbia's work. These portray a variety of infant forms in pleading postures, which surmount the pillars and occupy the wall veil between the spring of the arches. The range of low windows above the loggia suggests to the modern sanitarian a sacrifice of hygiene to architecture, and he unconsciously pictures to himself a correspondence between the internal arrangements for the infants with the mediæval exterior. But the director has requested me to be at the door at five o'clock, and I find him there with the Chef de Clinique, Dr. Bosi, to whose guidance I am entrusted. We pass under the main portico and enter a small door on the right. I soon learn that this small door is the Rubicon which divides mediæval surroundings from modern hygiene. "This is the receiving room," says Dr. Bosi. "Illegitimacy is the passport to our hospital, although we also admit the children of legally married parents who are recommended and maintained by the municipality. There are about 1,000 admissions annually. Each infant is brought here with a certificate from a medical man, stating the date of birth and all that can be ascertained about the health of the parents, especially as regards syphilis." It is at once washed and weighed, and is tended by a midwife in the adjacent waiting room pending the arrival of Dr. Bosi, or the second medical officer.

The first examination is very thorough, and should it in conjunction with the history as to the condition of the parents be satisfactory, the child is appointed to a "balia," or wet nurse, and consigned to the ward for healthy children. Should there be any suspicion of syphilis the infant is closely watched and periodically weighed to ascertain if its daily increase in weight is normal, and so long as any doubt exists as to hereditary taint it is artificially fed. Each infant on admission receives a card on which is entered the date of its birth, the sex, weight and general condition, and also its identifying number and letter. This card accompanies the individual throughout its life of adoption by the institute, viz.: till the age of 21, and furnishes a history of its organic life. At the same time an aluminium medallion, on which is stamped its identifying letter and number, is placed round its neck and fastened by crushing a small leaden bullet which runs on a cord necklet. For illegitimate children the cord is white and the medallion silver, for legitimate children the cord is blue and the medallion gilt.

The receiving and waiting rooms may be described as types of the wards throughout the establishment. Cleanliness, fresh paint, iron swing cots, and clean linen strike the eye at once. The angle between the wall and floor is filled in concave and, like the floor and walls to a height of two metres, it is varnished. Each ward has a cupboard for clean linen, a linen warming oven, and a trap-door in the wall, opening into a shaft, through which soiled linen finds its way to the basement. We now proceed upstairs, and pass through a large garret-like room into which the low windows seen from the piazza open, and which is destined for accessory purposes, not for the abode of the foundlings. These occupy the east aspect of the building, which is lighted by high windows overlooking the extensive garden and dairy farm. The numerous wards lead out of a broad and airy corridor, which on the opposite side is pierced by glazed eyelet holes, each one commanding an isolation ward, which are thus under the maternal eye of the sister in charge, while the entrance to them is quite apart.

After inspecting the wards we visited the medical officers' sanctum, well found in testing apparatus for all necessary purposes. Important among these are the various appliances for the daily testing of milk. The working hypothesis is, that if the proportion of cream in a sample of milk is adequate, the milk is of a sufficiently good quality. The microscope, and Conrad's apparatus combined, are the tests on which Dr. Bosi chiefly relies. After giving me a practical demonstration of testing by the last-named apparatus, we pass on to the incubators for weakly and prematurely-born infants. The inner or warmer chamber is maintained at a temperature ranging between 20° and 25° C. Should the temperature pass beyond these limits in either direction, a bi-metallic spring thermometer rings a warning bell in the sisters' room. A hygrometer indicates the degree of moisture in the chamber. The outer chamber is kept at a temperature ranging from 18° to 20° C. and has similar appliances for regulating the heat and moisture. It serves to break the sudden change from the high temperature of the warmer chamber to that of the ordinary wards, which are maintained at 15° or 16° C. Dr. Bosi finally showed me the garden and playground, and the movable slab by means of which the heifers are turned on their side for inoculation or for abstracting lymph.

The infants are kept in hospital for a minimum of 15 days, the healthy ones are then sent to foster-mothers in the country, while the diseased or suspected ones are kept under observation or treatment. The foster-parents receive 10 francs a month during the first year of charge, 8 francs during the second, and 6 francs during the third, fourth and fifth years. At five years of age the child can make itself useful in the podere (farm), and its foster-parents are usually willing to keep it on permanently. It would appear that when the foundlings grow up they are in no wise ashamed of their connection with the institution which adopted them; on the contrary, they sometimes adopt it as a patronyme; thus a baker who supplies bread to a goodly number of English families is known as "Baldassare degli Innocenti."

On bidding farewell to my courteous cicerone, I carried away the impression that many a child of wealthy parents would stand a better chance under the roof of the "Innocenti" than under the indulgent eye of its natural mother.

